# The overlooked pillars of medical education: addressing the mental health and well-being of medical school administrative staff

**DOI:** 10.3389/fpsyt.2025.1634323

**Published:** 2025-11-05

**Authors:** Jonathan Shaw, Charles Lai, Peter Bota, Jonathan Townsend

**Affiliations:** ^1^ California University of Science and Medicine, School of Medicine, Colton, CA, United States; ^2^ California University of Science and Medicine, Educational Operations, Colton, CA, United States

**Keywords:** burnout, medical education, mental health, administrative staff, medical school

## 1 Introduction

Medical schools function as intricate systems composed of various interdependent roles, including faculty, students, leadership, and administrative staff. While a significant amount of literature has explored the well-being, mental stress, and professional development of students and clinical staff, the administrative workforce has received comparatively little scholarly attention. This oversight persists despite the fact that administrative staff are deeply embedded in every foundation of a medical school’s infrastructure. These foundations include: admissions, curriculum support, coordination with outside hospitals, compliance, human resources, and beyond. Without their work, the medical institutions cannot effectively and efficiently function.

Administrative staff face many occupational pressures: high workloads, constrained authority, lack of professional recognition, and role conflict/ambiguity (1). These challenges contribute to chronic stress, job dissatisfaction, and burnout. Moreover, the onset of the COVID-19 pandemic intensified these conditions, as the transition to remote work, increased bureaucratic demands, and systemic instability placed unprecedented pressure on non-clinical employees. Despite this, the research on how these pressures impact administrative staff, especially within medical education, is sparse. This commentary reviews the limited literature on the mental health, workload, and organizational well being of administrative personnel in medical schools, advocating for their inclusion in institutional wellness initiatives and scholarly attention.

## 2 Administrative staff and burnout

Burnout is a well-documented adverse mental health outcome which can occur in workplaces, characterized by exhaustion, depersonalization, and reduced personal accomplishment (2). While burnout is frequently studied among clinicians, it is increasingly recognized in non-clinical roles, including administrative staff. An Iranian study assessed the administrative workload and its correlation with burnout among administrative staff and concluded that high workloads were significantly associated with emotional exhaustion and declining performance (3). The study emphasized that administrative staff in healthcare institutions operate under pressure comparable to that of clinical staff and often with little access to supportive resources (3). Similarly, another Iranian study evaluated burnout in healthcare and administrative personnel and found high levels of emotional strain, albeit healthcare staff scored significantly higher in emotional exhaustion than their administrative peers (4). The study underscored the need for institutions to monitor administrative staff’s occupational health, particularly in environments where staff responsibilities increase without proportional authority or acknowledgment (4).

### 2.1 The administrative workforce during the COVID-19 pandemic

The COVID-19 pandemic was a significant moment for all sectors of healthcare and education, exposing both weaknesses and strengths in institutional infrastructure. This was evident in a 2022 study which examined Hawaiian medical school faculty and staff well-being in the Fall of 2020 using a modified version of the Higher Education Data Sharing Consortium COVID-19 Institutional Response Staff and Faculty survey instruments (5). Administrative staff, in particular, experienced heightened levels of stress and uncertainty due to unclear expectations, rapid policy shifts, and blurred work-life boundaries during remote operations (5). Meanwhile, a study conducted across five oncology institutions in Bosnia and Herzegovina that utilized the DASS-21 scale to assess depression, anxiety, and stress in healthcare and administrative staff indicated high levels of psychological distress across both groups during the pandemic’s peak (6). Similarly, a 2022 Italian study reported alarming levels of anxiety and depression among university administrative staff, suggesting that this workforce endured the pandemic’s mental health burden on par with students and faculty, yet remained absent from institutional support discussions (7). These studies collectively signal that administrative staff are vulnerable to systemic disruptions such as pandemics and crises, due to their vital but often invisible role in maintaining institutional continuity (5–7).

### 2.2 Relational coordination and workplace dynamics

While much of the literature documents negative psychological outcomes, some research explores protective factors and workplace dynamics that buffer against burnout. A 2022 study of a medical school in the United Arab Emirates that assessed relational coordination among students, faculty, and administrative staff during the pandemic found that effective interdepartmental communication and mutual respect improved perceptions of mental health and job satisfaction (8). In settings where administrative staff felt integrated into decision-making processes and valued by academic counterparts, psychological outcomes were more favorable (8).

Some studies have applied the Job Demands-Resources (JD-R) model to analyze how job demands and resources shaped the mental health of administrative staff at Italian public universities during the COVID-19 return-to-work phase (9). It was found that while increased workload and ambiguity strained the administrative staff, the presence of personal resources, such as mental health resources and organizational support, mitigated adverse outcomes (9). These findings align with the JD-R model’s central tenet—that burnout emerges not merely from excessive demands, but from an imbalance between demands and accessible resources (9). The JD-R model was also used to examine primary care clerical staff in the U.S. Department of Veterans Affairs system. It was concluded that administrative burnout is not unique to academic settings but that it is pervasive across sectors where clerical labor is undervalued and overstretched (10).

### 2.3 Cross-cultural insights and global relevance

Burnout and workplace stress are not confined to any single country or region. A 2021 Malaysian study conducted a large-scale survey among administrative staff at a Malaysian public university and revealed widespread symptoms of depression, anxiety, and stress (11). Similarly, a 2009 United Arab Emirates study of the cognitive states of medical students and staff found a high prevalence of psychological distress, especially among support staff that lacked access to mental health services (12). These international studies reveal a common pattern: administrative staff across diverse sociocultural contexts face elevated psychological risks, compounded by limited institutional recognition. They also suggest the need for tailored mental health frameworks that consider cultural nuances, especially in globalized educational institutions (11, 12).

## 3 Difficulties in comparing results between countries and regions

The existing literature on well-being in medical school administrative staff is varied both in geographical settings but also in what measures are used to quantify wellness. Of the literature examined in this commentary, studies from Asia, the Middle East, Europe, and the United States were referenced. The lack of standardized measures made it difficult to compare these studies, for example multiple studies used the MBI (1, 3, 4, 10) or the DASS-21 (6, 11). In terms of measuring burnout, the MBI is the golden standard for the literature, but other measures such as the Oldenburg Burnout Inventory (9) or modifications of the MBI (10) were also used in the studies included in this commentary. The measurement scales used for mood disorders, such as anxiety and depression, displayed even more variation, with the references using various measures like the DASS-21, Patient Health Questionnaire-9, General Anxiety Disorder-7, Beck Depression Inventory, and Beck Anxiety Inventory (6, 7, 11, 12). This heterogeneity of measurements in the literature results in difficulties in making direct comparisons between the results of studies conducted in different geographical regions or countries. To resolve this, the use of standardized definitions, criteria, and measures should be adopted by the literature for future studies as discussed below.

## 4 Towards better measurement and intervention

One major barrier to improving administrative staff well-being is the absence of reliable, role-sensitive assessment tools. While the Maslach Burnout Inventory (MBI) remains the most widely used instrument, some literature have raised concerns that the MBI may have reliability issues when applied to certain populations, as exemplified by the case of a pilot study conducted in Nigerian resident physicians which found the MBI’s Cronbach alpha ranged from 0.62 to 0.82 while the Copenhagen Burnout Inventory (CBI) ranged from 0.83 to 0.91 depending on which subsection was examined (2). A 2021 study in the United States also validated the CBI among nurses, indicating its potential adaptability to administrative contexts (13). The CBI’s distinction between personal, work-related, and client-related burnout dimensions may also provide a more nuanced assessment of administrative stressors (2). Developing and standardizing such tools is crucial for early detection, policy development, and targeted intervention. Moreover, disaggregating data by staff role within institutions will allow researchers and policymakers to identify at-risk groups and design tailored wellness programs. The selection of a standardized, free to use, and publicly available measure such as the CBI can also allow for institutions in countries with limited resources to contribute to the literature while also obtaining useful data for local quality improvement programs in their institutions.

## 5 The indispensable role of administrative staff

Despite limited scholarly focus, the importance of administrative staff in medical education is undeniable. These professionals ensure institutional compliance, manage student and faculty services, support accreditation, maintain curriculum infrastructure, and facilitate communication among diverse departments. Without them, the academic engine of a medical school grinds to a halt. Unfortunately, this essential role is often rendered invisible to researchers by the ambiguous nature of the term, “staff,” with many publications focusing on teaching faculty or physicians instead of administrative staff. Additionally, many administrative workers operate behind the scenes and thus do not interact directly with students and faculty, further limiting their visibility. This invisibility can contribute to occupational dissatisfaction, fuel attrition, and undermine institutional foundation. Recognizing administrative staff as integral stakeholders in medical education is both an ethical obligation and a strategic necessity. Investing in their well-being through inclusive policies, mental health resources, professional development, and acknowledgment could ensure stronger and more cohesive institutions which will benefit all of its community members.

## 6 Conclusion

Medical school administrative staff constitute a vital, yet understudied segment of the academic workforce. Despite their importance to institutional operations, their experiences of stress, burnout, and workplace dissatisfaction in this population often go overlooked in the literature. The current literature reviewed in this manuscript affirms their vulnerability, especially during and in the aftermath of crises like the COVID-19 pandemic. The literature also highlights potential protective factors such as relational coordination and supportive resources. To promote sustainable and efficient educational environments, medical schools must advocate for wellness research to include not only faculty and students but also administrative staff. We must embrace a holistic approach that acknowledges the administrative workforce—not merely as support staff, but as essential stakeholders of the academic mission. Only then can we build institutions that value every contributor, foster genuine resilience, and advance equity in academic medicine.
